# Green Synthesis, Formulation and Test Field of *Lymantria monacha* L. (Lepidoptera: Lymantriidae) Sex Pheromone in East European Region

**DOI:** 10.3390/ijms26020568

**Published:** 2025-01-10

**Authors:** Iuliana Vasian, Mihai-Leonard Duduman, Emese Gal, Adrian Pîrnău, Monica Gorgan

**Affiliations:** 1 Pheromone Production Center, “Raluca Ripan” Institute for Research in Chemistry, “Babes-Bolyai” University, 30 Fantanele Street, 400294 Cluj-Napoca, Romania; monica.bucsa@ubbcluj.ro; 2 Applied Ecology Laboratory, Forestry Faculty, “Stefan cel Mare” University of Suceava, 13 University Street, 720229 Suceava, Romania; mduduman@usv.ro; 3 Faculty of Chemistry and Chemical Engineering, “Babes-Bolyai” University, 11 Arany Janos Street, 400028 Cluj-Napoca, Romania; emese.gal@ubbcluj.ro; 4 National Institute for Research and Development of Isotopic and Molecular Technologies, 67-103 Donath Street, 400293 Cluj-Napoca, Romania; adrian.pirnau@itim-cj.ro

**Keywords:** *Lymantria monacha*, (±)-disparlure, (±)-monachalure, (*Z*)-2-methyl-7-octadecene, test field

## Abstract

The nun moth, *Lymantria monacha* L. (Linnaeus, 1758), is one of the most important defoliators of coniferous forests in Europe and Asia. In sexual communication, females produce three epoxides and an alkene: (−)-disparlure [(7*S*,8*R*)-*cis*-7,8-epoxy-2-methyloctadecane], (+)-monachalure [(7*R*,8*S*)-*cis*-7,8-epoxyoctadecane], (−)-monachalure [(7*S*,8*R*)-*cis*-7,8-epoxyoctadecane], and their corresponding olefins. This study aimed to develop a green synthesis pathway for all pheromonal components, emphasizing the use of common raw materials, a simplified three-step process, lower costs, and an environmentally friendly approach compared to existing methods. The proposed method introduces a novel synthetic route employing an innovative improvement alkylation step catalyzed by lithium iodide in diglyme. The synthesized compounds were characterized using GC-MS, ^1^H NMR, and ^13^C NMR spectroscopy. Six synthetic blends were formulated for field testing, with the goal of identifying the most attractive composition. Field trials demonstrated that a blend with a 10:10:1 ratio of (±)-Disparlure, (±)-Monachalure, and (*Z*)-2-Methyl-7-octadecene exhibited the highest attraction efficiency.

## 1. Introduction

The nun moth (*Lymantria monacha* L., Lepidoptera: Erebidae) is a polyphagous insect pest species that feeds on the leaves of coniferous and broadleaved trees in Europe and Asia. It is an extremely dangerous defoliator, especially for spruce, pine, fir, larch, and Douglas fir [1]. Spring defoliation by larvae can kill affected trees, particularly conifers, leading to extensive losses despite intervention with biological and chemical control measures [2].

The discovery of disparlure (*cis*-7,8-epoxy-2-methyloctadecane) as the sex pheromone of the gypsy moth (*Lymantria dispar*) by Bierl et al. in 1970 marked a major milestone in lepidopteran chemical ecology. This compound was initially isolated from extracts of 78,000 abdominal segment tips of female moths [3,4]. Subsequent research revealed that the sex pheromones of the *Lymantria* genus are more chemically diverse than previously recognized. For example, the precursor molecule, *cis*-olefin, was also identified in the same extracts and shown to possess attractant properties for various *Lymantria* species (Gries 1996, 1997, 2005, Morewood 1999) [5,6,7,8]. The sex pheromone blend of the nun moth was later characterized to include (−)-disparlure [(7*S*,8*R*)-*cis*-7,8-epoxy-2-methyloctadecane], (+)-monachalure [(7*R*,8*S*)-*cis*-7,8-epoxyoctadecane], (−)-monachalure [(7*S*,8*R*)-*cis*-7,8-epoxyoctadecane], and (*Z*)-2-methyl-7-octadecene.

Numerous methods for synthesizing disparlure have been developed. The earliest synthesis, reported by Bierl et al. and Eiter in 1972, involved the preparation of an appropriate olefin followed by epoxidation. However, this approach exhibited poor stereoselectivity, necessitating extensive purification [3,4,9]. In 1974 and 1976, Bestmann et al. proposed a more stereoselective method, beginning with isoamyl bromide to synthesize isooctyltriphenylphosphonium bromide. The corresponding ylide was reacted with undecanal in a Wittig reaction to produce (*Z*)-2-methyl-7-octadecene, which was subsequently epoxidized with *m*-chloroperbenzoic acid to yield disparlure [10,11].

Chan et al. (1974, 1985) developed an alternative synthesis using a trialkylsilylallyl anion, which underwent regio- and stereoselective alkylation with alkyl halides to produce γ-products with *trans*-stereochemistry at the double bond. These *trans*-vinylsilanes were then converted into *Z*-vinyl iodides, which were coupled with organometallic reagents to form *Z*-alkenes. Epoxidation of the *Z*-alkenes yielded disparlure [12,13]. Other methods have been reported by Iwaki et al. (1974) and Farnum et al. (1977) [14,15].

Although the synthesis of disparlure has been thoroughly investigated, with numerous methods reported in the literature [16,17,18,19,20,21,22,23,24,25,26,27,28,29,30,31,32,33,34,35], the synthesis of monachalure has been comparatively underexplored, with only a few studies dedicated to this subject [36].

The population dynamics of *L. monacha* are monitored throughout the forested areas of Romania, with a focus on spruce stands and mixed forests containing beech, using atraLYMON pheromone traps. These traps are provided by the Pheromone Production Center at the Institute for Research in Chemistry, Babes-Bolyai University. To improve plant and environmental protection, synthesis methods for semiochemicals are continuously updated [37,38,39].

Until 2002, in Romania, the synthetic pheromone bait used for monitoring *L. monacha* contained only (±)-disparlure, synthesized according to the method described by Botar et al., which employed metallic lithium and liquid ammonia (NH₃) as starting materials [32]. After 2003, (±)-disparlure, along with additional components such as (±)-monachalure (*cis*-7,8-epoxyoctadecane) and (*Z*)-2-methyl-7-octadecene, were synthesized in our laboratory using organomercuric compounds as intermediates [38,39]. However, these methods were found to be highly toxic to both the environment and human health. According to the classification in Directive 67/548/EEC and 1999/45/EC on toxicity, mercury is classified as T+ (very toxic), T (toxic), and N (dangerous for the environment) (see MSDS). To address these concerns, we sought to develop alternative methods that are more environmentally friendly and less harmful while maintaining excellent results. In this study, we describe an improved alkylation method that employs lithium iodide (LiI) as a catalyst in diglyme. This process is designated as a “green synthesis” due to its clean, safe, cost-effective, and environmentally friendly characteristics. To validate the functionality of the synthesized compounds, we conducted field tests alongside NMR and GC-MS analyses, comparing their efficacy with that of the commercial product atraLYMON.

**Figure 1:**
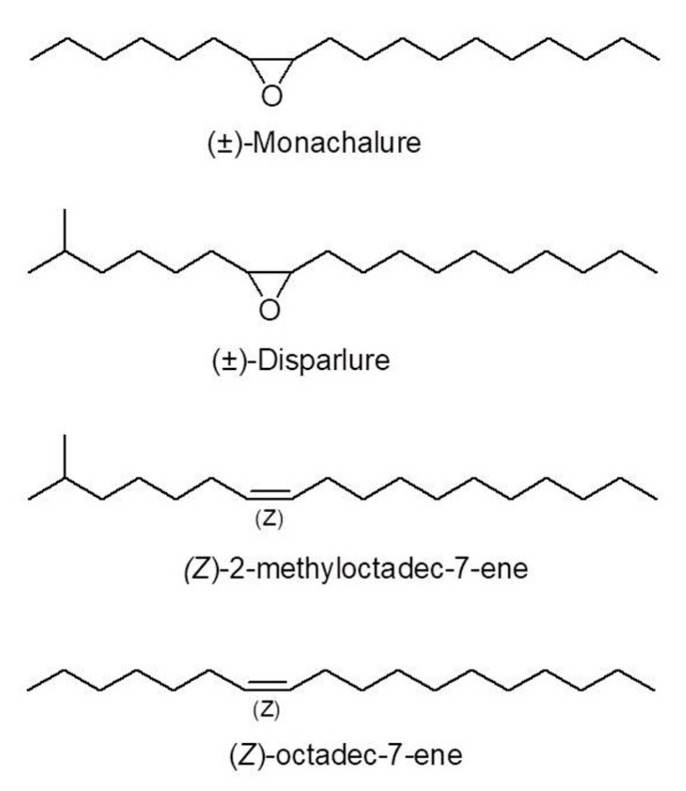
Chemical structure of *Lymantria monacha* synthetic sex pheromone components.

Gries et al. identified the composition of the nun moth sex pheromone as (+)-monachalure, (+)-disparlure, and (*Z*)-2-methyl-7-octadecene in a 10:10:1 ratio [8]. The decision to synthesize and utilize racemic disparlure was based on practical and experimental considerations. Racemic disparlure is easier and more cost-effective to synthesize compared to the enantiomerically pure compound. Moreover, previous studies (e.g., [Botar et al., Oprean et al.]) [37,38,39] have shown that racemic disparlure demonstrates sufficient biological activity to attract *L. monacha* moths in field applications. These studies indicate that the stereoisomeric purity of disparlure, while potentially enhancing specificity in some contexts, is not critical for achieving effective attraction in practical applications. By using racemic disparlure, we were able to simplify the synthesis, reduce costs, and focus on testing the efficacy of various pheromone blends under field conditions. This approach aligns with the goals of our study, which aimed to optimize synthesis and evaluate pheromone blends in a practical and scalable manner. Also, the presence of (−)-disparlure and (−)-monachalure had no effect on attraction of *L. monacha* (Gries et al., 1996) [5]. Thus, the other developed pheromone lure for *L. monacha* detection surveys in North America (Morewood et al., 1999) [7], containing (±)-disparlure, (±)-monachalure and 2me-Z7-18Hy at a 20:20:1 blend remains suitable for the attraction of *L. monacha* from both Central Europe and Japan (Gries et al., 1997) [6]. In our study, we used (±)-monachalure, (±)-disparlure, (*Z*)-2-methyl-7-octadecene, and (*Z*)-7-octadecene in various formulations [6,7] (Figure 1).

## 2. Results

### 2.1. Synthesis of Pheromonal Components

**Scheme 1:**
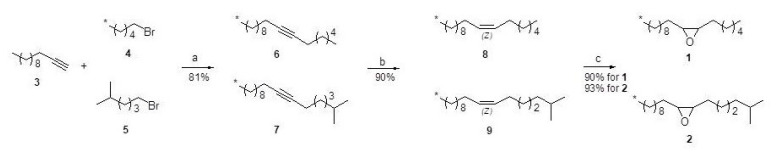
Synthesis method of compounds: (±)-disparlure (**1**) and (±)-monachalure (**2**). Conditions: (a) *n*-BuLi/diglyme (−50 °C)/LiI (−10 °C)/bromine **4** or **5**, 90–110 °C; (b) H_2_, NiP_2_/Ethanol, rt; (c) HCOOH/H_2_O_2_, rt. * Abbreviated structural formula in ChemDraw.

We have developed an innovative improved alkylation method to synthesize all components of the nun moth sex pheromone (*L. monacha*). Our approach is based on a C6 or C7 + C12 strategy, involving an original coupling reaction with lithium iodide as a catalyst in diglyme (Scheme 1). This method allowed us to avoid the use of mercury-based organometallic compounds previously employed in pheromone production [37,38,39]. The reactions proceeded with favorable yields, and the mercury-free synthesis has the dual benefit of reducing both the environmental and health impacts of chemical pheromone production, thereby contributing to the advancement of green chemistry technologies.

Alkynes **6** and **7** were hydrogenated following Brown’s method [40], yielding highly stereoselective alkenes **8** and **9** (as determined by GC, compared with the Wiley Spectral Libraries) with significant yields of 90%. To avoid using *meta*-chloroperbenzoic acid (MCPBA), we performed the epoxidation using the Gamage method, which employs formic acid and hydrogen peroxide [41]. This approach produced (±)-disparlure (**1**) and (±)-monachalure (**2**) with yields of 90% for **1** and 93% for **2**, and overall yields of 66% and 68%, respectively (Scheme 1).

Our laboratory focuses on synthesizing semiochemical compounds used in plant protection. Accordingly, we performed additional couplings to obtain intermediates required for the synthesis of various compounds with insect-attracting activity, as presented in Table 1. We also extended our alkylation method to other substrates to evaluate its reproducibility (Table 1).

It was observed that the coupling reactions involving substrates with functional groups at the R-Br position (entries 1–12) exhibited significantly higher yields > 91% compared to those with inactivated groups (entries 13–16), with only 81–86% maximum obtained (Table 1). Additionally, we found that when R-OTs were used, the reaction did not occur. This result clearly indicates that lithium iodide functions exclusively as a catalyst and does not directly interact with any of the reactants when R-X compounds with bromine are used.

Table 2 presents the optimal results for the alkylation reactions of compounds 1–16, as described in Table 1. From a practical standpoint, we observed that, for bromides containing -OTHP or -O*t*Bu groups, using an excess of terminal alkyne (2 equivalents) facilitated the complete consumption of the bromide. The resulting crude product could easily be purified from the excess of alkyne through column chromatography. However, in the case of fully inactivated bromides, the use of excess alkyne did not yield the same effect, necessitating an excess of bromide, which was subsequently removed by high-vacuum distillation. This approach allowed us to obtain compounds with a GC purity > 98%. In our study, we also tested THF as a solvent, but the reaction yields did not exceed 50%, leading us to abandon this approach. We will continue working on improving this alkylation method because of its practical significance.

The intermediates and final products were characterized using conventional ^1^HNMR, ^13^C NMR, and GC-MS techniques; refs. [42,43,44,45,46,47,48,49,50,51,52,53,54,55] original spectra were listed in the Supplementary Materials for entry 15, 16 (S1–S18). Furthermore, field trials were conducted, employing pheromone traps to compare our results with the standard pheromone blend, atraLYMON, which is widely used in production.

### 2.2. Field Tests

Six variants of pheromonal blends (M1, M2, M3, M4, M5, and M6) were developed, with M1 representing the atraLYMON production lure and M2–M6 comprising novel blends proposed for the attraction of *L. monacha* males (Table 3).

**Figure 2:**
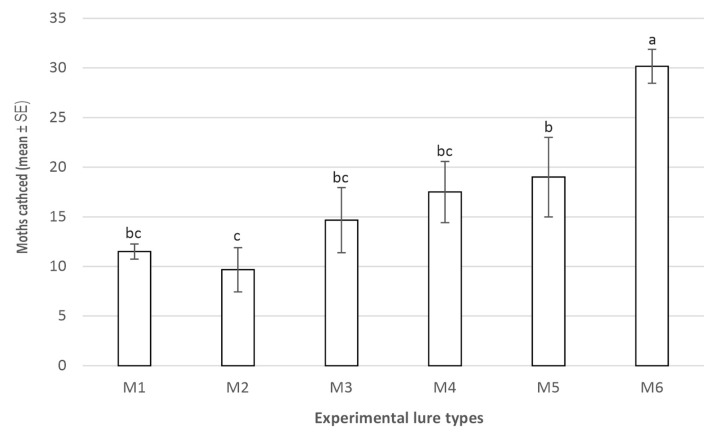
Comparison of *L. monacha* males caught in the field tests for traps baited with the six experimental lure types (bars with different lower-case letters indicate significant differences, according to Tukey’s multiple comparison test: *p* < 0.05).

During the field tests, a total of 615 adult *L. monacha* males were captured. The highest number of individuals was recorded with the experimental lure M6 (30.2 ± 4.2 moths per trap), while the fewest were captured with lure types M2 (9.67 ± 5.5 moths per trap) and M1 (control) (11.5 ± 1.8 moths per trap). The catches with M6 were significantly higher than those with all other tested lures (Figure 2), whereas the catches with the other lures did not differ significantly from the control (M1) (one-way ANOVA: n = 36; DF = 5; F = 14.9487; *p* < 0.0001). The average number of catches compared to the commercial lures (M1) ranged from −6.1% (M3) to +162.3% (M6).

The results indicate that variants M3, M4, M5, and M6 exhibited higher attractiveness compared to the control (M1). However, the attractiveness of variants M3 and M4 was lower than that of variant M6, possibly due to the presence of (*Z*)-7-octadecene, which appears to exert a negative influence on the pheromone bait. This inhibitory effect is most pronounced in variant M2, which demonstrated the lowest level of attractiveness. Further experimental studies are necessary to elucidate the precise role and impact of (*Z*)-7-octadecene on the efficacy of the pheromone bait.

## 3. Discussion

The formation of carbon–carbon bonds through the classical alkylation of metal acetylides with alkyl halides has traditionally been achieved using lithium or sodium in liquid ammonia [36,56]. However, this method is challenging due to the difficulty of handling liquid ammonia and solubility issues arising from the use of acetylides. Alternative reaction conditions have been developed, such as the use of hexamethylphosphoramide (HMPA) as a dipolar aprotic solvent [57]. Unfortunately, HMPA poses significant health risks as a carcinogen, having been shown to cause nasal cancer in rats [58].

In 1988, N,N-dimethylpropyleneurea (DMPU) was introduced as a safer and equally effective alternative to HMPT [59]. Dimethyl sulfoxide (DMSO) has also been used, but its acidity restricts its application to relatively acidic alkynes, such as PhC≡CH and THPOCH_2_C≡CH [60]. Buck and Chong proposed an alkylation method that employs tetrahydrofuran (THF) as the solvent, enabling the alkylation of virtually any 1-alkyne with excellent results; however, this method is limited to 1-alkynes [61,62].

Maruyama et al. reported the regioselective coupling of lithium acetylides with allylic halides mediated by lithium iodide [63]. Hanko et al. demonstrated similar reactions, coupling but-3-yn-1-yl tetrahydropyranyl ether with *n*-octyl bromide and propynol tetrahydropyranyl ether with l-[4-bromobutyl]-4-ethyl-2,6,7-trioxabicyclo[2.2.2]octane in the presence of catalytic lithium iodide [64]. Razdan et al. further reported the coupling of terminal alkynes with benzylic bromides in THF under similar catalytic conditions [65]. These methods, employing activated halides, achieved yields ranging from 20% to 72% [61,62,63,64,65].

**Figure 3:**
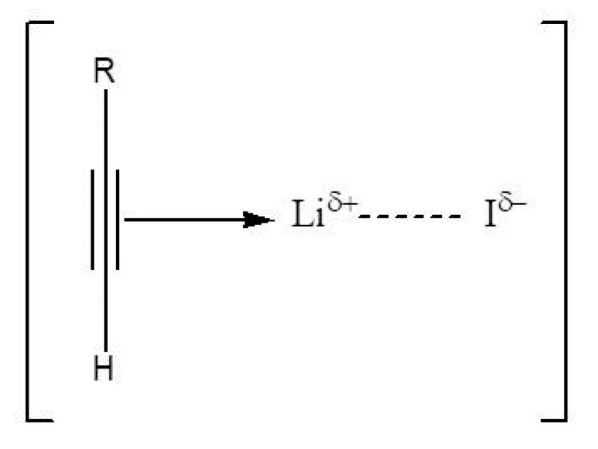
The supposed interaction of LiI with triple bond.

We attempted to apply these alkylation methods, but in our case, the reaction yield was only about 50%. We hypothesize that in our system, LiI may have the ability to polarize the triple bond, thereby facilitating formation anions ≡C^−^Li^+^, and to finally couple with bromide in diglyme (Figure 3) [66].

In the future, we plan to investigate this remarkable alkylation reaction using other halogen derivatives, such as X = Cl, I, F, or MgX.

Field tests demonstrated that the M6 variant, based on Gries studies, exhibited significantly greater attractiveness compared to the previously used production variant, atraLYMON (M1). This result indicates that our synthesis method is effective and that the compounds produced are viable. Furthermore, it was observed that the racemic mixture can be successfully incorporated into the pheromone blend for the monitoring of *L. monacha* populations.

Future studies will focus on developing new variants of pheromone blends to gain a deeper understanding of the role and impact of individual enantiomers in the attractant mixture.

In this paper, we focused on the synthesis method for all compounds, employing an innovative improvement alkylation technique and re-evaluating the synthesis pathways for monachalure and disparlure.

Behavioral observations confirmed that the compounds produced using our method exhibit a high attraction capacity, comparable to that of the classic version.

## 4. Materials and Methods

### 4.1. Reagents

All reactions were conducted under an argon atmosphere (Linde, Timisoara, Romania) in completely dry setups. For the alkylation step, cooling was achieved using an ethanol–liquid nitrogen bath (INCDTIM, Cluj-Napoca, Romania). Solvents used were purchased from Merck (Hamburg, Germany) and were of synthesis grade.

Reaction progress was monitored by thin-layer chromatography (TLC) on a Merck DC Alufolien, silica gel 60 F254, with component visualization performed using a 20% H_2_SO_4_ solution in ethanol.

The following reagents were employed without further purification: 1-dodecyne (98%, ACROS Organics, Belgium, WI, USA), 5-methyl-1-bromohexane (BLD PHARMATECH GmbH, Hamburg, Germany), 1-bromohexane (Merck, Sigma-Aldrich, Darmstadt, Germany), and diglyme (diethylene glycol dimethyl ether, Merck, Sigma-Aldrich, Darmstadt, Germany).

### 4.2. Spectral Measurements

All intermediates and reaction products were analyzed by GC-MS and NMR spectroscopy.

Agilent GC-MS Gas Chromatograph-7890A/5975/2008 (Agilent Technologies, Inc. Europe, Waldbronn, Germany) was used for analysis; GC-MS analyses were performed in scan mode on a DB-5MS (30 m × 0.25 mm × 0.25 µm) capillary column (Agilent 19091S-433M), with high purity He carrier gas at a flow rate of 1 mL/min.

^1^H-NMR (400 MHz) and ^13^C-NMR (101 MHz) spectra were recorded at room temperature in CDCl_3_ on a Bruker Avance 400 MHz spectrometer, using the solvent line as reference.

### 4.3. Field Tests

In addition to NMR and GC-MS analyses, we conducted field studies to observe the insects’ reactions to the substances we synthesized. By creating variants of specific pheromone blends, we allowed the insects themselves to provide the answers through their behavior, which ultimately surpasses any conventional analytical method.

The field tests were conducted in a 70–100-year-old spruce forest in the northern part of the Eastern Carpathians (Suceava County, Vama Forest District, Paltinu II Forest Unit (coordinates)). On 22 July 2013, 36 sticky traps (30/40 cm white adhesive panel) were set up, divided into six experimental blocks. Supplementary Materials, Figure S1. Each of the six traps in a block was baited with an experimental lure (Table 2). The traps were placed on tree trunks at a height of 2 m above the ground. The distance between two traps within a block was at least 50 m. The minimum distance between two experimental blocks was 300 m.

The experimental lures were prepared at the “Raluca Ripan” Institute for Research in Chemistry at “Babeș-Bolyai” University, Cluj-Napoca. Red rubber septa (19 mm) were loaded with the desired semiochemical variant of the lure in a 50 μL *n*-hexane solution containing 0.1% BHT (butylated hydroxytoluene) for field trials. After loading, the solvent was allowed to evaporate in a hood at room temperature for 30–45 min. The lures were then wrapped in aluminum envelopes and stored in a refrigerator until they were ready to be deployed in the field.

The attractiveness of the experimental lures was monitored at three weeks, until August 15, when the captured male moths were counted and the traps were cleaned.

Analysis of the differences between means of *L. monacha* male captures for each experimental lure type was performed using one-way ANOVA, after previously checking the normality (Shapiro–Wilk test) and homogeneity (Levene’s test). The significance of the differences was tested with the Tukey (HSD) procedure [67]. Testing of the significance of differences, homogeneity, and normality of distributions was performed using XLSTAT-PRO (version 2012) (Addinsoft, New York, NY, USA) plugged into Excel.

The moths were identified by the Laboratory of Entomology at Stefan cel Mare University in Suceava, Romania. The method demonstrated high specificity, as no significant non-target insects were captured.

### 4.4. Experimental Part

7-Octadecyne (**6**) and 2-Methyl-7-octadecyne (**7**): to a cold (−50 °C), stirred solution of 1-dodecyne (**3**) (8.4 g, 50.6 mmol) in diglyme (168 mL), 32 mL of *n*-BuLi (1.6 M in hexanes, 50.6 mmol) were added drop-wise and the mixture was kept at this temperature for 30 min. The solution was allowed to warm to −10 °C, and was treated with 6.88 g LiI (55.6 mmol, 1.1 eq.), then heated to 50 °C before dropwise addition of alkyl halide 61 mmol, 1.2 eq. (10 g bromide **4**, or 10.8 g bromide **5**). The reaction mixture was heated to 105–110 °C and stirred until all of the alkyl halide was consumed (ca. 4 h), and was then cooled to room temperature. Standard aqueous work-up was made (extraction with petroleum ether and washed with water and brine provided crude material). Purification was made by distillation of volatiles at low pressure, and flash chromatography, giving 10.25 g **6** and 10.85 g **7**, (>98% GC purity) with 81% yield. Supplementary Materials, Figure S2.

7-Octadecyne (**6**)

MS: (EI, 70 eV), *m*/*z* (I_rel_, %): M^+^ 250(<1), 221(1), 207(<1), 180(1), 165(9.7), 152(4.3), 137(3.9), 123(14.5), 109(49.5), 95(88.3), 81(100), 67(92.2), 55(58.2), 41(60.1) Supplementary Materials, S1. ^1^H NMR (400 MHz, CDCl_3_) δ: 2.15 (t, *J* = 6.9 Hz, 4H,C**H**_2_-C≡), 1.5–1.2 (m, 24H, -C**H**_2_-), 0.90 (td, *J* = 6.8, 3.3 Hz, 6H, CH_3_-) Supplementary Materials, S7. ^13^C NMR (101 MHz, CDCl_3_) δ 80.2 (2C), 31.9, 31.4, 29.6, 29.3, 29.1, 28.8, 28.5(2C), 22.7(2C), 22.6(2C), 18.7(2C), 14.0(2C) Supplementary Materials, S8.

2-Methyl-7-octadecyne (**7**)

MS: (EI, 70 eV), *m*/*z* (I_rel_, %): M^+^ 264(<1), 249(2.9), 221(1.9), 208(<1), 194(<1), 179(1.9), 165(3), 152(3.8), 137(6.8), 123(53.4), 109(61.1), 95(91.2), 81(100), 67(85.4), 55(62.1), 41(63.1) Supplementary Materials, S4. ^1^H NMR (400 MHz, CDCl_3_) δ: 2.15 (t, *J* = 6.1 Hz, 4H, C**H**_2_-C≡), 1.2–1.6 (m, 22H, -C**H**_2_-), 1.1–1.2 (m, 1H, -C**H**-), 0.89 (t, *J* = 6.1 Hz, 9H, -C**H**_3_) Supplementary Materials S13. ^13^C NMR (101 MHz, CDCl_3_) δ 80.18, 80.1, 38.4, 31.9, 29.6, 29.3(2C), 29.2(2C), 28.8, 27.9, 26.6, 22.7, 22.5(2C), 18.7(2C), 14.0(2C) Supplementary Materials S14.

*(Z)-7-Octadecene (***8***)* and *(Z)-2-Methyl-7-octadecene (***9***)*. The hydrogenation reaction was conducted with hydrogen at room temperature and atmospheric pressure in a device designed for that purpose. The installation was composed of a burette with a three-way valve filled with hydrogen, a water tank to maintain pressure in the burette, a hydrogen bottle, a flow regulator, a magnetic stirrer, and a Schlenk flask. First, Ni(OAc)_2_·4H_2_O (1.3 g, 5.1 mmol) was dissolved in ethanol 95% (41 mL) while stirring (eventually with slight heating), then a solution of NaBH_4_ (0.208 g, 5.4 mmol) in ethanol 95% (5.12 mL) was added under a hydrogen current, resulting in the formation of the NiP_2_ catalyst. The reaction mixture then heated up and turned black. Ethylenediamine (0.84 mL, 13.6 mmol) was added dropwise, and the stirring was stopped. Compound **6** (10.25 g, 41 mmol) or **7** (10.85 g, 41 mmol) was added in one portion and the hydrogen stream was shut. All the external valves were closed, the valve from the burette was opened and the stirring was started until no hydrogen consumption was observed (theoretical consumption 918 mL H_2_ depending on the temperature and the atmospheric pressure). Then, the valve of the burette was closed and the flow-regulating valve was opened. The reaction mixture was diluted with diethyl ether (100 mL) and filtered on a G4 filter funnel. The filtrate was washed successively with water and aq. HCl to a neutral pH, and was finally brined, dried over anhydrous MgSO_4_, and concentrated by a rotary evaporator. The residue was purified by flash column chromatography silica gel (*n*-Hexane) to give **8** (9.3 g) or **9** (9.8 g), (>98% GC purity) with 90% yield. Rf = 0.96. Supplementary Materials, Figure S3.

(*Z*)-7-Octadecene (**8**)

MS: (EI, 70 eV), *m*/*z* (I_rel_, %): M^+^ 252(36.8), 273(<1), 224(2.9), 210(<1), 196(<1), 182(1.4), 168(2.9), 154(3.8), 139(7.7), 125(25.2), 111(58.2), 97(100), 83(99), 69(93.2), 55(61.1), 41(63.1) Supplementary Materials, S1.^1^H NMR (400 MHz, CDCl_3_) δ: 5.39 (t, *J* = 4.7 Hz, 2H, -C**H**=), 2.1–2.0 (m, 4H, -C**H**_2_-CH=), 1.34 (d, *J* = 24.3 Hz, 24H, -C**H**_2_-), 0.9–1.0 (m, 6H, -C**H**_3_) Supplementary Materials, S9. ^13^C NMR (101 MHz, CDCl_3_) δ 129.8(2C), 31.9, 31.8, 29.8, 29.7(2C), 29.6(2C), 29.4, 29.3, 29.0, 27.2(2C), 22.7(2C), 14.1(2C) Supplementary Materials, S10.

(*Z*)-2-Methyl-7-octadecene (**9**)

MS: (EI, 70 eV), *m*/*z* (I_rel_, %): M^+^ 266(13.5), 151(<1), 238(2.4), 223(<1), 210(1.9), 195(<1), 182(1), 167(<1), 153(1), 139(2.9), 125(10.6), 111(31), 97(53.4), 83(64), 69(88.3), 56(100), 43(68.9) Supplementary Materials, S5. ^1^H NMR (400 MHz, CDCl_3_) δ: 5.38 (t, *J* = 4.7 Hz, 2H, -C**H**=), 2.09–2.01 (m, 4H, -C**H**_2_-CH=), 1.61–1.49 (m, 1H, -C**H**-), 1.36–1.26 (m, 22H, -C**H**_2_-), 1.24–1.16 (m, 3H, -C**H**_3_), 0.91 (dd, *J* = 9.2, 6.6 Hz, 6H,-C**H**_3_) Supplementary Materials, S15. ^13^C NMR (101 MHz, CDCl_3_) δ 129.9(2C), 38.9, 31.9, 31.6, 30.0, 29.8, 29.7(2C), 29.6, 29.4, 29.3, 28.0, 27.2, 27.1, 22.7, 22.6, 14.1(2C) Supplementary Materials, S16.

(*±*)-*Monachalure* (**1**) and (*±*)-*Disparlure* (**2**).

A known amount of 100% formic acid (3.7 g, 80 mmol) 2.2 eq. was placed in a 250 mL flask containing the required amount of alkene **8** (9.3 g, 37 mmol) or **9** (9.8 g, 37 mmol). The mixture was cooled to a temperature of 10 °C, and the required amount of 30% hydrogen peroxide (35.4 g, 312 mmol), previously equilibrated at 10 °C, was added dropwise with continuous stirring for about 1 h. Thereafter, the temperature of the reaction mixture was raised to 60 °C and maintained at this temperature for a period of 8 h. The reaction mixture was cooled to room temperature and was diluted with 100 mL diethyl ether. The organic layer was washed with water until acid free, dried over sodium sulfate and concentrated by a rotary evaporator. The residue was purified by flash column chromatography with silica gel (*n*-Hexane:Et_2_O = 20:1) to give (±)-Monachalure (**1**) (8.9 g) or (±)-Disparlure (**2**) (9.7 g), (>98% GC purity) with 90% and 93% yields, respectively; Rf = 0.76.

(±)-Monachalure, *cis*-7,8-Epoxyoctadecane (**1**)

MS: (EI, 70 eV), *m*/*z* (I_rel_, %): M^+^ 268(<1), 250(<1), 239(<1), 225(<1), 211(<1), 197(<1), 183(6.8), 169(<1), 152(1.9), 138(<1), 127(9.7), 109(6.8), 97(42.7), 83(36.8), 69(57.2), 55(100), 43(84.4) Supplementary Materials, S3. ^1^H NMR (400 MHz, CDCl_3_) δ: 2.90 (t, *J* = 4.3 Hz, 2H, -C**H**-O-), 1.56–1.26 (m, 28H, C**H**_2_-), 0.94–0.84 (m, 6H, -C**H**_3_) Supplementary Materials, S11. ^13^C NMR (101 MHz, CDCl_3_) δ: 57.2(2C), 31.9(2C), 31.8(2C), 29.5(2C), 29.3, 29.2(2C), 27.8(2C), 26.6, 22.7, 22.5, 14.0(2C) Supplementary Materials, S12.

(±)-Disparlure, *cis*-2-Methyl-7,8-epoxyoctadecane (**2**)

MS: (EI, 70 eV), *m*/*z* (I_rel_, %): M^+^ 282(<1), 264(<1), 253(<1), 239(<1), 225(<1), 211(<1), 198(<1), 183(6,8), 169(1), 152(1.9), 141(3.9), 123(4.8), 111(17.4), 97(33), 82(45.6), 69(88.3), 55(82.5), 43(100) Supplementary Materials, S6. ^1^H NMR (400 MHz, CDCl_3_) δ: 2.91 (p, *J* = 4.1 Hz, 2H, -C**H**-O-), 1.62–1.24 (m, 27H, C**H**_2_-), 1.24–1.15 (m, 3H, -C**H**_3_), 0.89 (t, *J* = 6.5 Hz, 6H, -C**H**_3_) Supplementary Materials, S17. ^13^C NMR (101 MHz, CDCl_3_) δ: 57.2(2C), 38.9, 31.9(2C), 29.6(2C), 29.3(2C), 27.8(2C), 27.3, 26.8, 26.5, 22.6(2C), 22.5, 14.0(2C) Supplementary Materials, S18.

## 5. Conclusions

A new synthesis method for all the pheromonal components of the *Lymantria monacha* lepidopteran sex pheromone was developed, employing a minimal number of steps, good overall yields, and the least toxic starting materials.

An innovative improvement alkylation method was utilized, using LiI as a catalyst in diglyme, achieving excellent yields. This method was successfully extended to the synthesis of other components of insect pheromones, yielding similarly promising results.

The compounds produced using our new method were tested in the field and demonstrated high efficacy as pheromonal lures.

Six attractive synthetic blends were developed for field testing to identify the composition with the highest attractiveness. Field tests revealed that the blend (±)-Disparlure:(±)-Monachalure:(*Z*)-2-Methyl-7-octadecene in a 10:10:1 ratio exhibited the greatest attraction capacity.

Future research will aim to develop novel pheromone blend variants to better understand the role and influence of individual enantiomers within the attractant mixture.

## Figures and Tables

**Table 1:** The alkylation technique employed in synthesizing certain intermediates, which we utilize in preparing active substances for pheromone production (yield of alkylation).

Entry	Primary Alkyne	R-X	Alkylation Product *n*-BuLi/Diglyme/LiI	Yield %	Semiochemical Compounds and Pest Species [*]
1	CH_3_-(CH_2_)_3_-C≡CH	*t*BuO-(CH_2_)_6_-Br	CH_3_-(CH_2_)_3_-C≡C-(CH_2_)_6_-O*t*Bu	91	E7-12 Ac or Z7-12 Ac [42] *Plusia gamma*, *Agrotis segetum*
2	CH_3_-(CH_2_)_3_-C≡CH	THPO-(CH_2_)_8_-Br	CH_3_-(CH_2_)_3_-C≡C-(CH_2_)_8_-OTHP	98	Z9-14 Ac [43] *Agrotis exclamationis*
3	CH_3_-(CH_2_)_3_-C≡CH	THPO-(CH_2_)_9_-Br	CH_3_-(CH_2_)_3_-C≡C-(CH_2_)_9_-OTHP	91	2E,13Z-18 Ac [44] *Zeuzera pyrina*
4	CH_3_-(CH_2_)_3_-C≡CH	THPO-(CH_2_)_10_-Br	CH_3_-(CH_2_)_3_-C≡C-(CH_2_)_10_-OTHP	93	Z11-16 Ald(Ac) [45] *Helicoverpa armigera*
5	CH_3_-(CH_2_)_3_-C≡CH	THPO-(CH_2_)_12_-Br	CH_3_-(CH_2_)_3_-C≡C-(CH_2_)_12_-OTHP	91	Z13-18 Ald [46] *Homoeosoma nebulellum*
6	CH_3_-(CH_2_)_4_-C≡CH	THPO-(CH_2_)_7_-Br	CH_3_-(CH_2_)_4_-C≡C-(CH_2_)_7_-OTHP	92	Z8-14 Ac [47] *Spilonota ocellana*
7	CH_3_-(CH_2_)_4_-C≡CH	THPO-(CH_2_)_10_-Br	CH_3_-(CH_2_)_4_-C≡C-(CH_2_)_10_-OTHP	96	Z11-17 Ac [48] *Mamestra brassicae*
8	CH_3_-(CH_2_)_5_-C≡CH	*t*BuO-(CH_2_)_6_-Br	CH_3_-(CH_2_)_5_-C≡C-(CH_2_)_6_-O*t*Bu	95	Z7-14 Ac [49] *Amathes c-nigrum*
9	CH_3_-(CH_2_)_5_-C≡CH	THPO-(CH_2_)_4_-Br	CH_3_-(CH_2_)_5_-C≡C-(CH_2_)_4_-OTHP	95	Z5-12 Ac [50] *Cossus cossus*
10	CH_3_-(CH_2_)_5_-C≡CH	THPO-(CH_2_)_8_-Br	CH_3_-(CH_2_)_5_-C≡C-(CH_2_)_8_-OTHP	98	Z9-16 Ald [51] *Helicoverpa armigera*
11	CH_3_-(CH_2_)_7_-C≡CH	*t*BuO-(CH_2_)_4_-Br	CH_3_-(CH_2_)_7_-C≡C-(CH_2_)_4_-O*t*Bu	98	Z5-14 Ac [52] *Cossus cossus*
12	CH_3_-(CH_2_)_7_-C≡CH	THPO-(CH_2_)_6_-Br	CH_3_-(CH_2_)_7_-C≡C-(CH_2_)_6_-OTHP	95	2-Acetoxy-8-heptadecene [53] *Dasineura gleditchiae*
13	THPO-CH_2_-C≡CH	CH_3_-(CH_2_)_4_-HC=CH-(CH_2_)_8_-OTs	No reaction	-	2E,13Z-18 Ac [54] *Zeuzera pyrina*
14	THPO-(CH_2_)_2_-C≡CH	CH_3_-(CH_2_)_5_-Br	THPO-(CH_2_)_2_-C≡C- (CH_2_)_5_-CH_3_	86	Z3-10 Ac [55] *Cossus cossus*
15	CH_3_-(CH_2_)_9_-C≡CH	CH_3_-(CH_2_)_5_-Br	CH_3_-(CH_2_)_9_-C≡C-(CH_2_)_5_-CH_3_	81	(±)-Monachalure [38] *Lymantra monacha*
16	CH_3_-(CH_2_)_9_-C≡CH	(CH_3_)_2_-CH-(CH_2_)_4_-Br	CH_3_-(CH_2_)_9_-C≡C-(CH_2_)_4_-CH-(CH_3_)_2_	81	(±)-Disparlure [6] *Lymantria monacha* *Lymantria dispar*

* References for characterization of semiochemical compounds.

**Table 2:** Conditions of the reaction for the best alkylation method yields in this study.

Entry	Primary Alkyne (Equiv.)	R-X (Equiv.)	LiI (Equiv.)	Yield %
1–12	2	1	1.1	>91
13	2	1	1.1	-
14	1	1.2–1.5	1.1	86
15, 16	1	1.2–1.5	1.1	81

**Table 3:** Experimental lures used in the field tests.

Experimental Lure Variants	Chemical Composition
M1 (commercial lure atraLYMON)	1 mg (±)-Disparlure
M2	1 mg (±)-Monachalure + 0.1 mg (*Z*)-7-octadecene
M3	0.5 mg (±)-Monachalure + 0.5 mg (±)-Disparlure + 0.05 mg (*Z*)-7-octadecene
M4	0.5 mg (±)-Monachalure + 0.5 mg (±)-Disparlure + 0.05 mg (*Z*)-7-Octadecene + 0.05 mg (*Z*)-2-Methyl-7-octadecene
M5	0.5 mg (±)-Monachalure + 0.250 mg (±)-Disparlure + 0.05 mg (*Z*)-2-Methyl-7-octadecene
M6	0.5 mg (±)-Monachalure + 0.5 mg (±)-Disparlure + 0.05 mg (*Z*)-2-Methyl-7-octadecene

## Data Availability

Data is contained within the article and Supplementary Materials.

## Supplementary Materials

The following supporting information can be downloaded at https://www.mdpi.com/article/10.3390/ijms26020568/s1.
