# Supplementary material for: Green Synthesis, Formulation and Test Field of Lymantria monacha L. (Lepidoptera: Lymantriidae) Sex Pheromone in East European Region
Source: Int J Mol Sci. 2025 Jan 10;26(2):568. doi: 10.3390/ijms26020568 (PMC11766351; doi:10.3390/ijms26020568)
Supplement: Supplementary file 1 [file ijms-26-00568-s001.zip › ijms-3372071-supplementary.pdf]

Supporting Information

For

Green Synthesis, Formulation and Test Field of *Lymantria monacha* L. (Lepidoptera: Lymantriidae) Sex Pheromone in East European Region

Iuliana Vasian [1,*], Mihai-Leonard Duduman [2], Emese Gal [3], Adrian Pîrnău [4] and Monica Gorgan [1]

[1] Pheromone Production Center, "Raluca Ripan" Institute for Research in Chemistry, "Babes-Bolyai" University, 30 FantaneleStreet, 400294 Cluj-Napoca, Romania; monica.bucsa@ubbcluj.ro
[2] Applied Ecology Laboratory, Forestry Faculty, "Stefan cel Mare" University of Suceava, 13 University Street, 720229 Suceava, Romania; mduduman@usv.ro
[3] Faculty of Chemistry and Chemical Engineering, "Babes-Bolyai" University, 11 Arany Janos Street, 400028 Cluj Napoca, Romania; emese.gal@ubbcluj.ro
[4] National Institute for Research and Development of Isotopic and Molecular Technologies, 67-103 Donath Street, 400293 Cluj-Napoca, Romania; adrian.pirnau@itim-cj.ro
* Correspondence: iuliana.vasian@ubbcluj.ro; Tel.: +40-264-580165

Table of contents

GC-MS spectra of all compounts S1-S6

NMR spectra of all compounds S7-S18

Pheromone trap used Figure S1

The Alkylation installation Figure S2

The Hydrogenation installation Figure S3

GC-MS spectra of all compounts

7-Octadecyne (6), S1

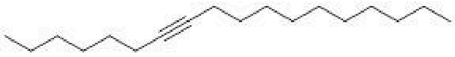

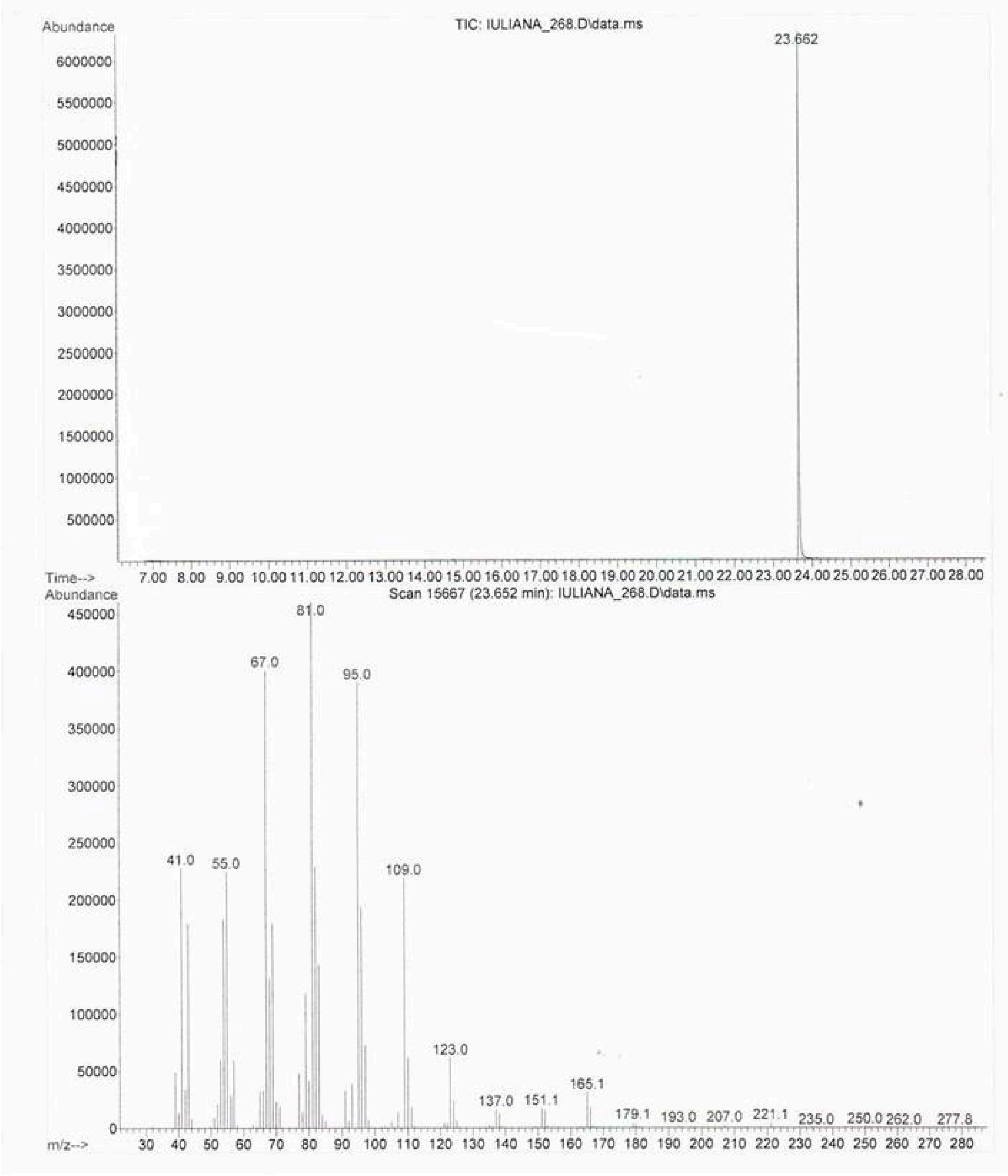

Z-7-Octadecene (8), S2

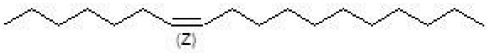

(Z)-octadec-7-ene

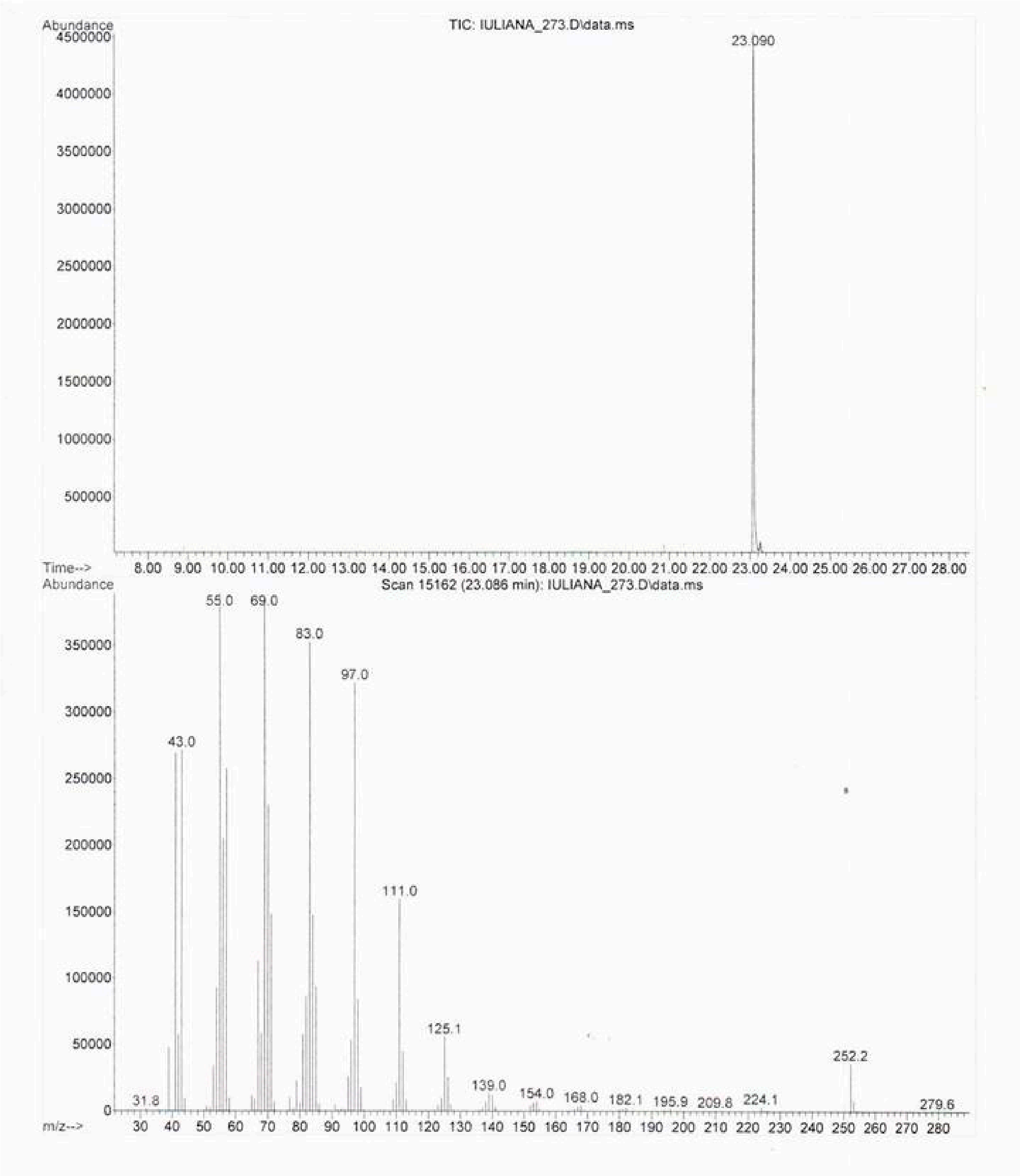

(±)-Monachalure (1), S3

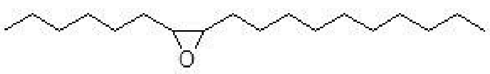

(±)-Monachalure

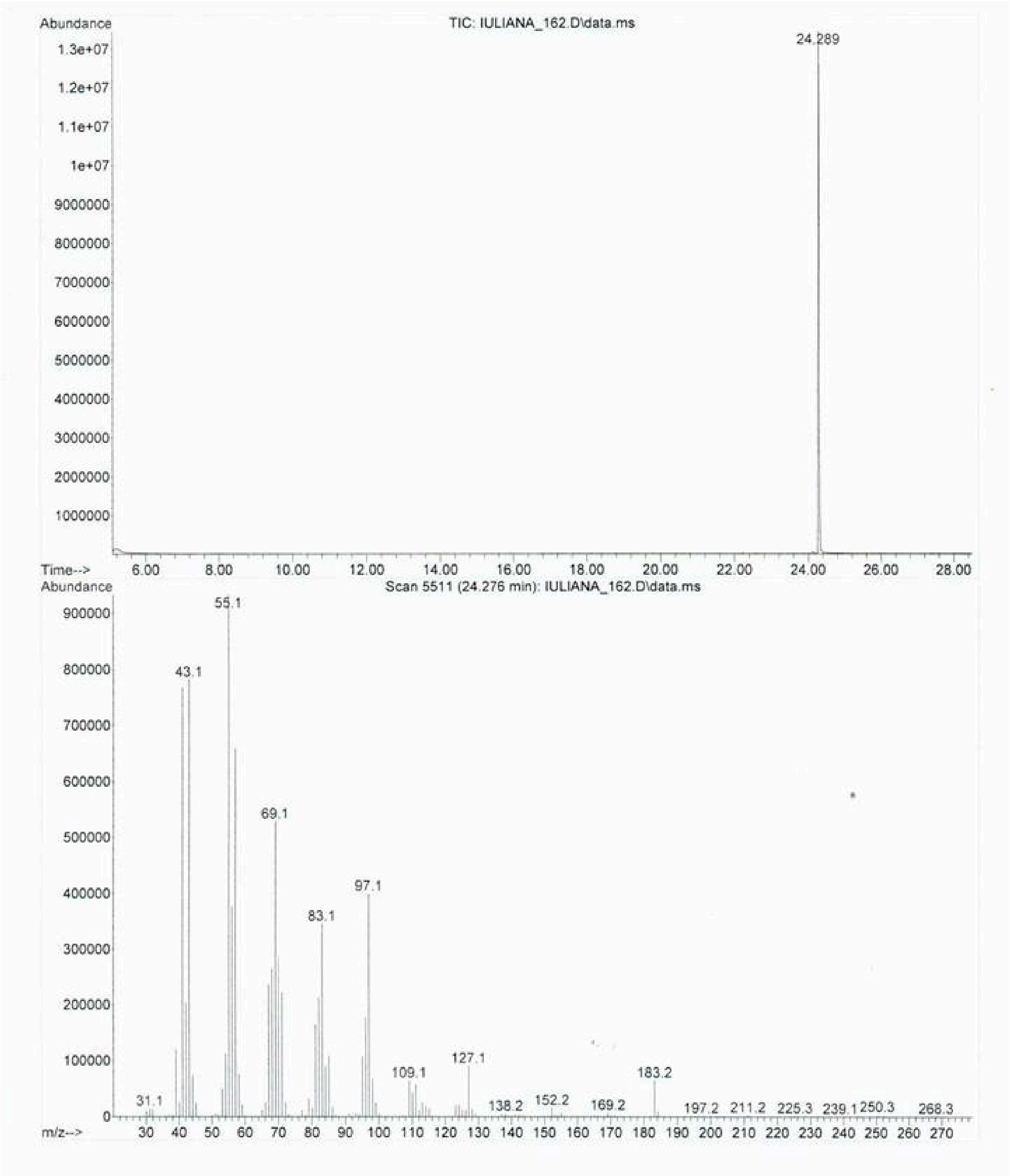

2-Methyl-7-octadecyne (7), S4, * Abbreviated structural formula in ChemDraw.

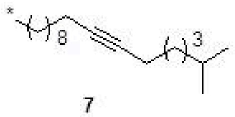

7

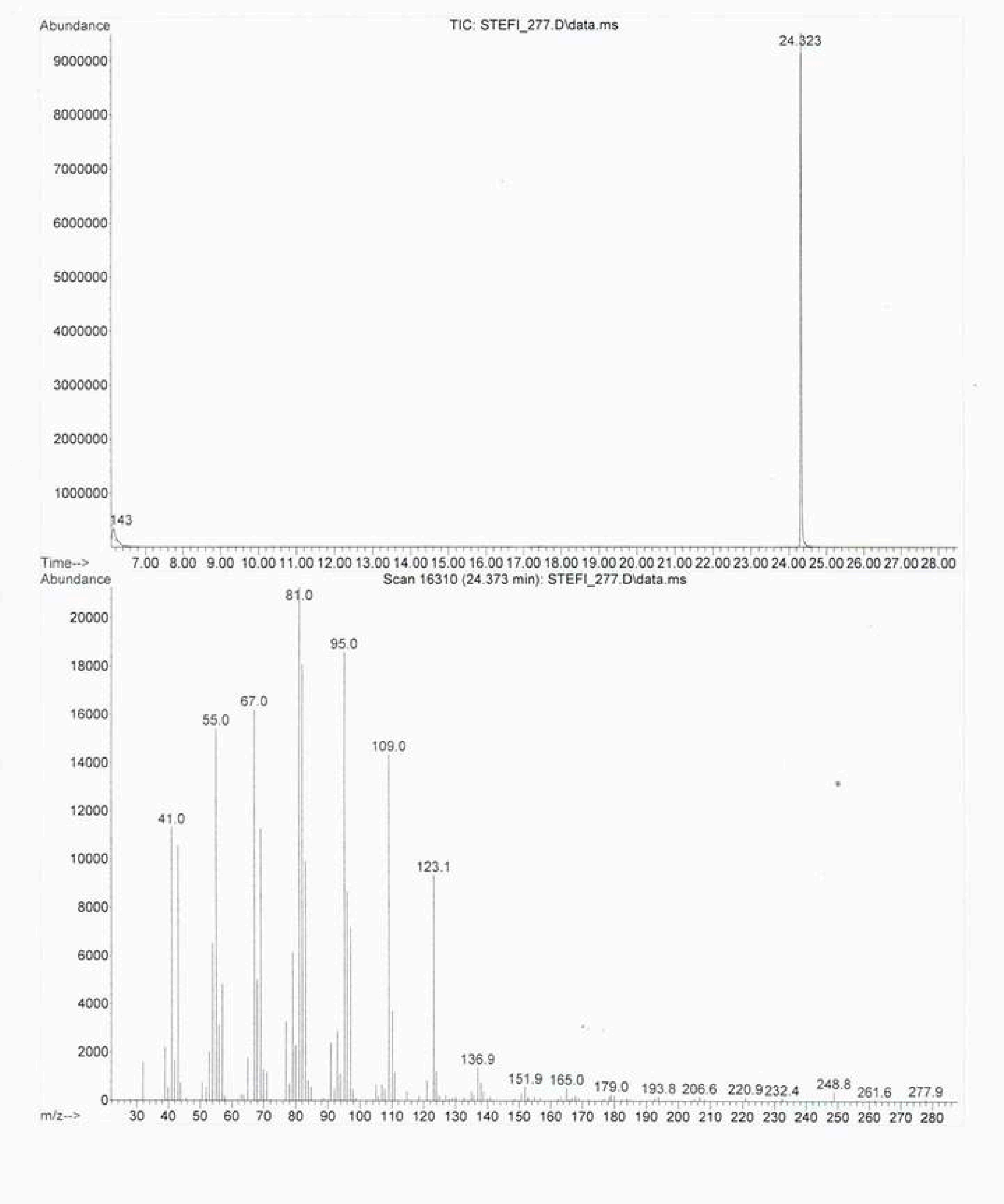

Z-2-Methyl-7-octadecene (9), S5

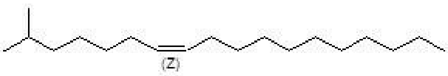

(Z)-2-methyloctadec-7-ene

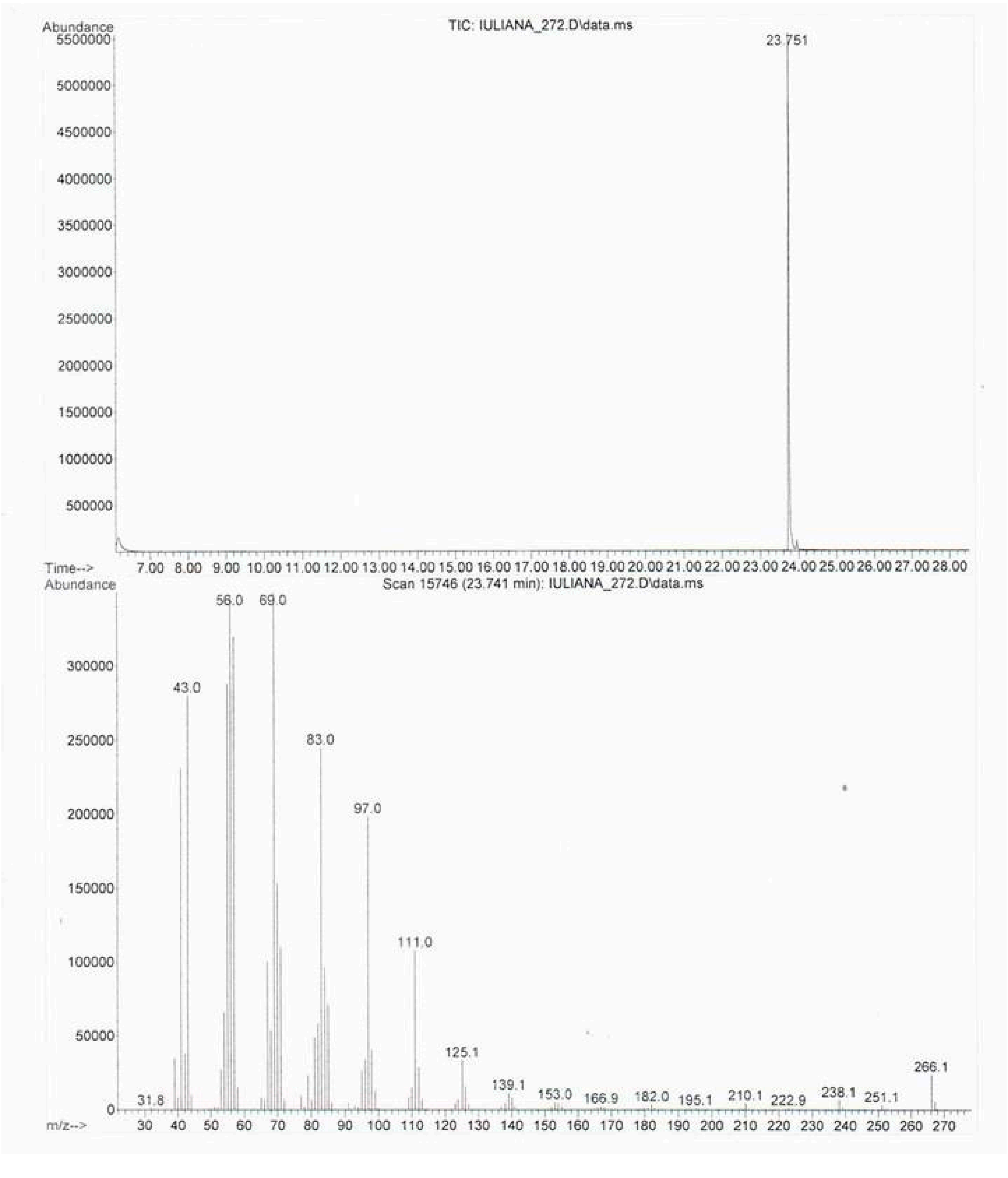

(±)-Disparlure (2), S6

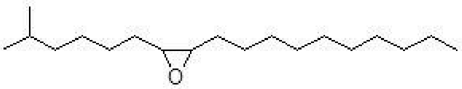

(±)-Disparlure

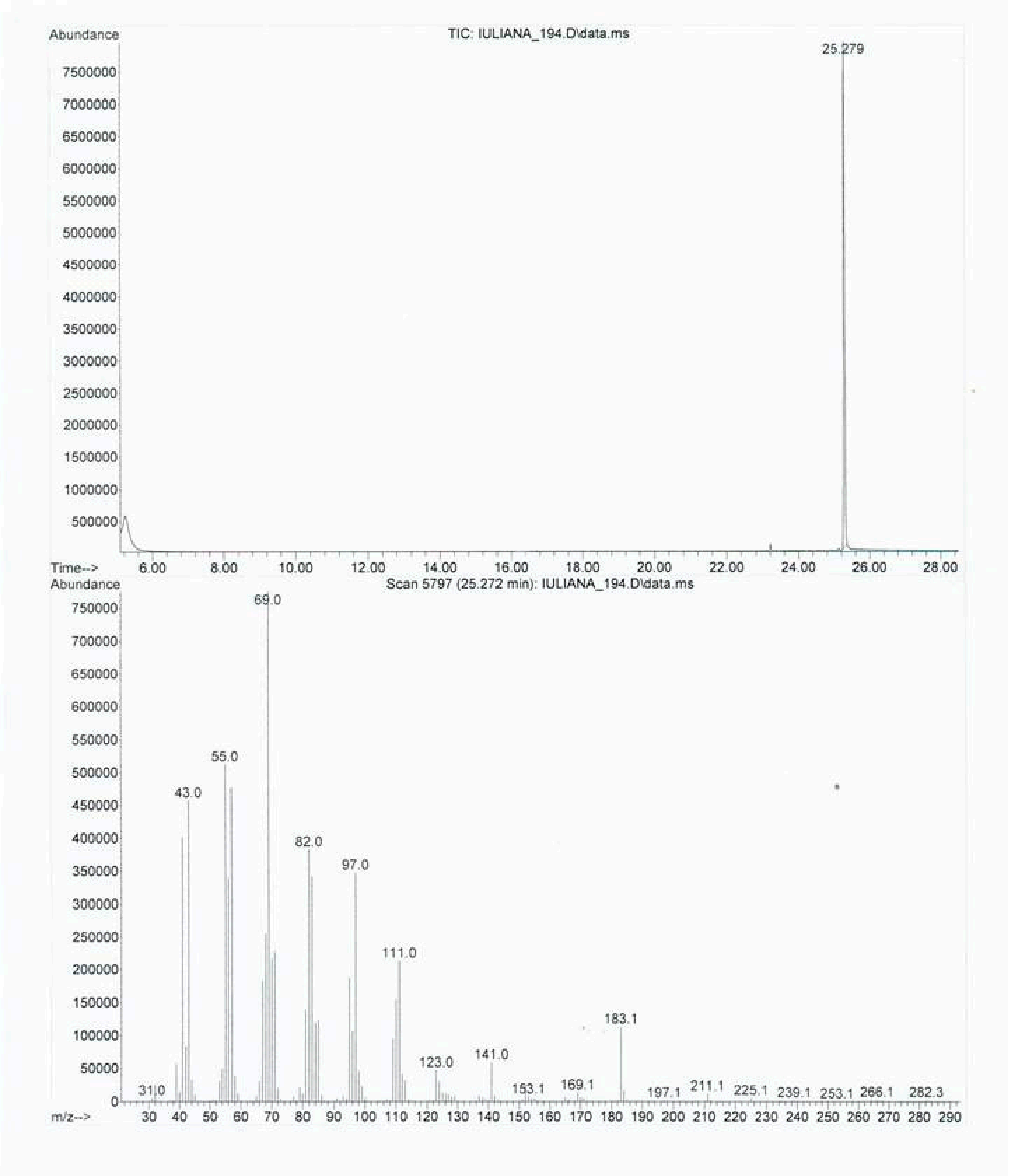

NMR-Analysis

7-Octadecyne (6), S7 ^{1}H-NMR, S8 ^{13}C-NMR, * Abbreviated structural formula in ChemDraw.

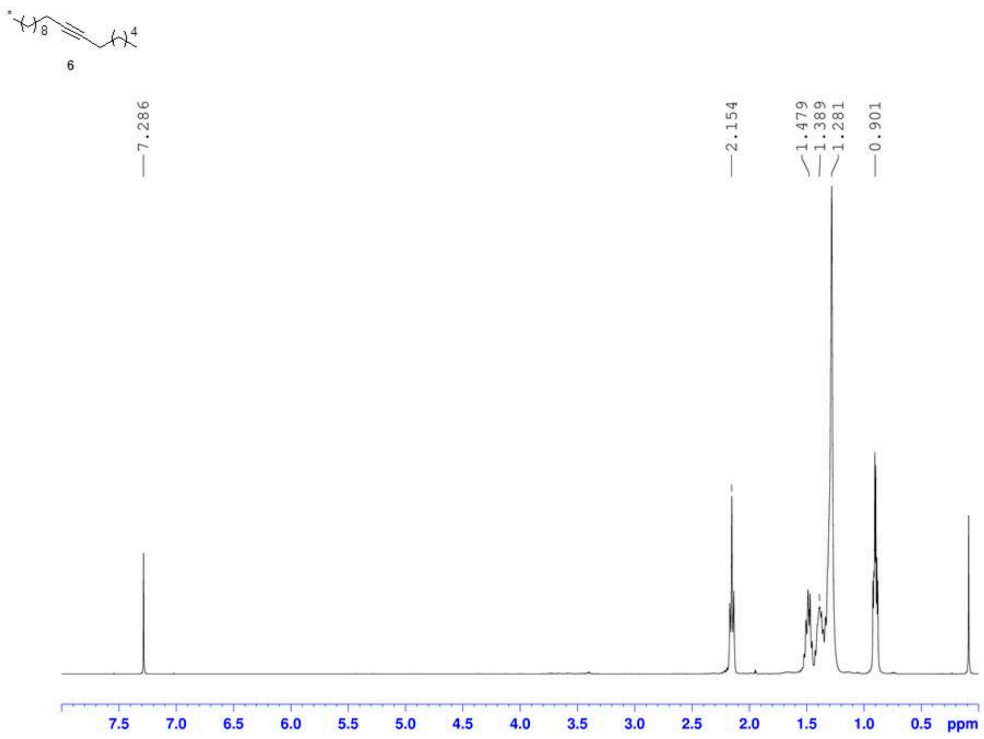

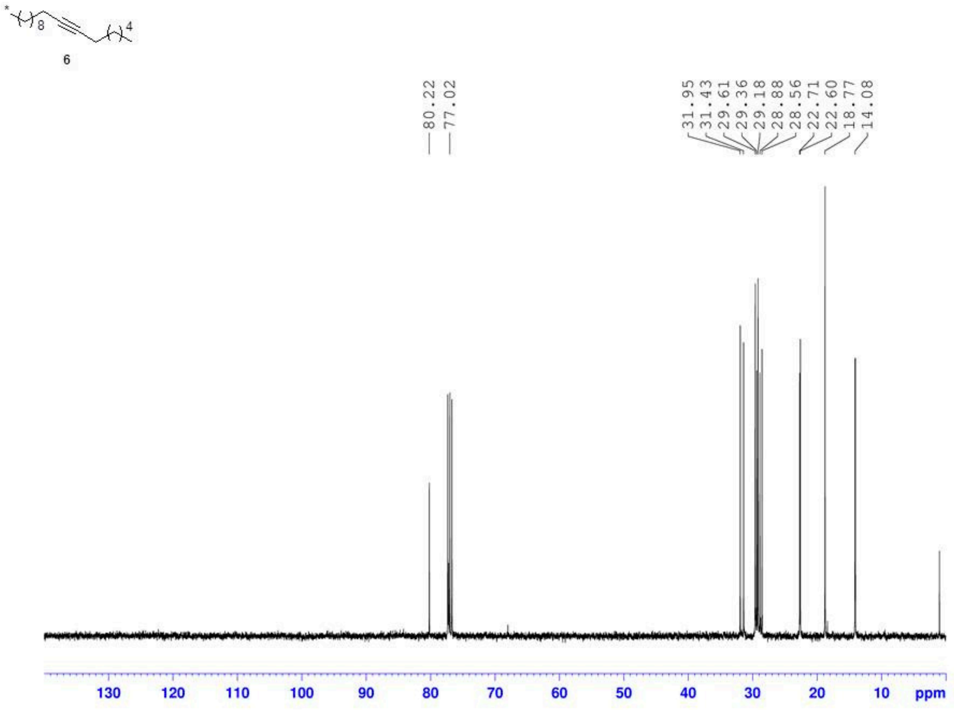

(*Z*)-7-Octadecene(8), S9 ¹H-NMR, S10 ¹³C-NMR

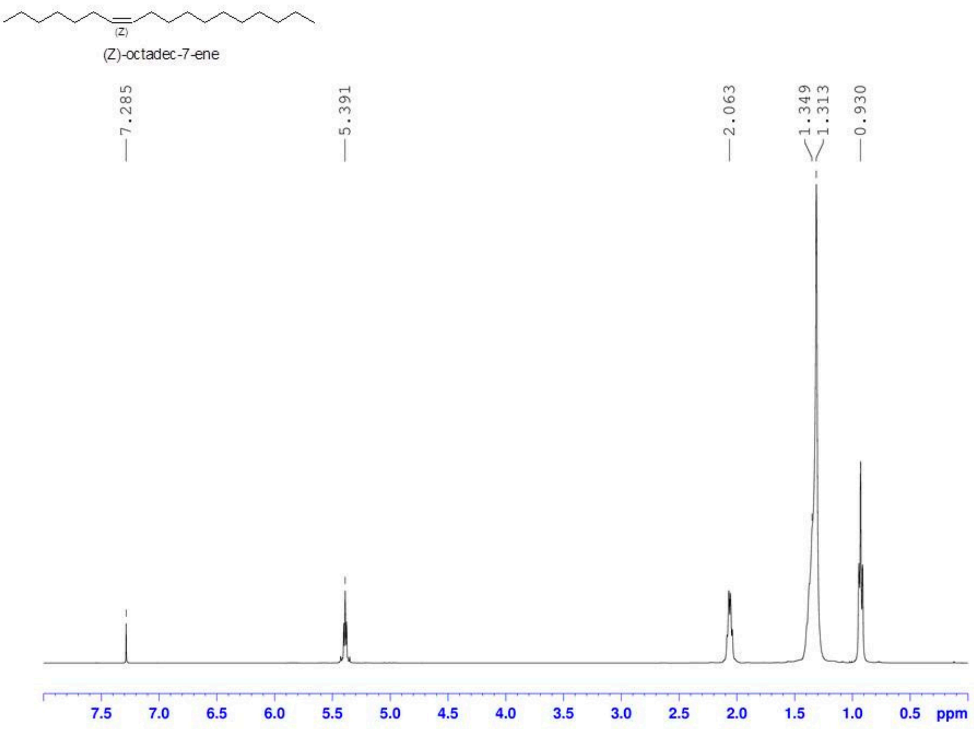

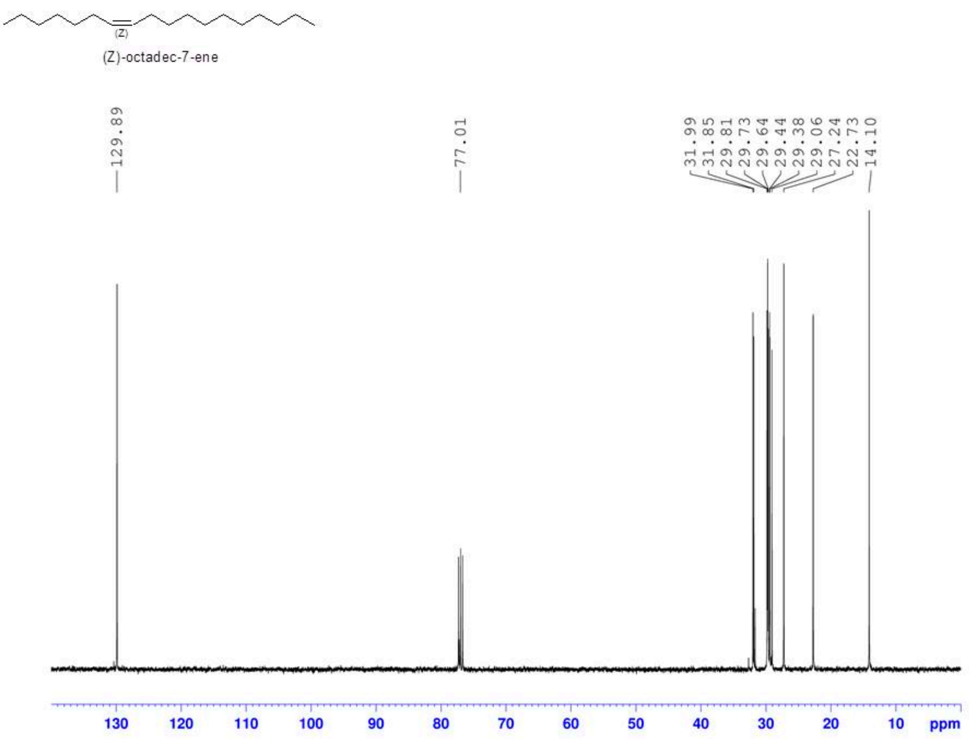

(±)-Monachalure (1), S11 ¹H-NMR, S12 ¹³C-NMR

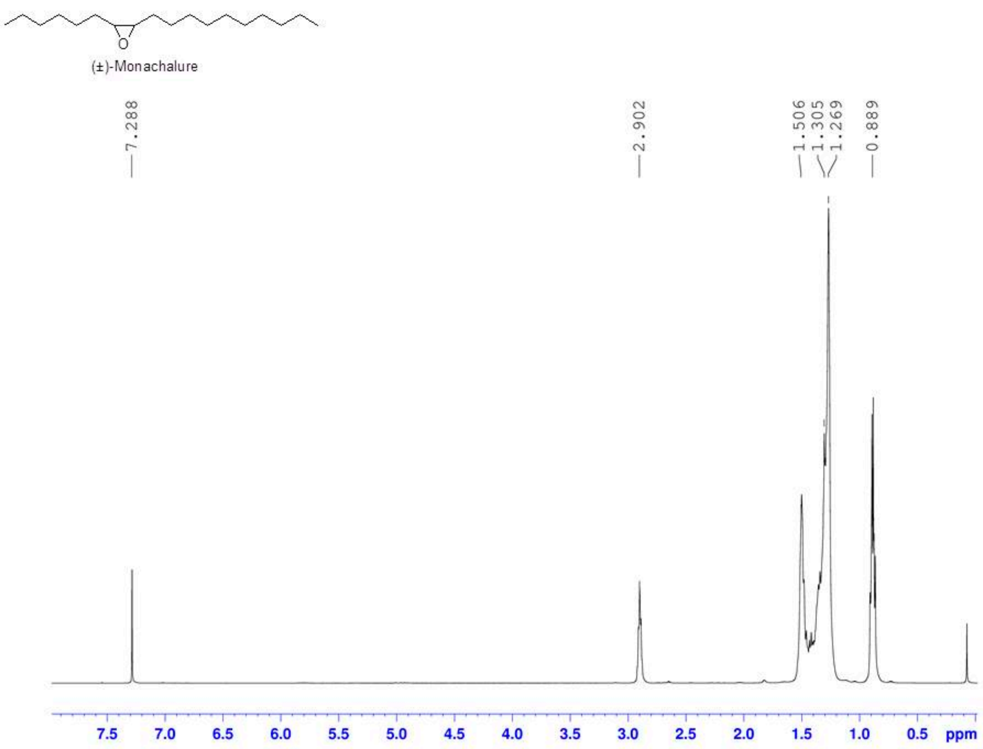

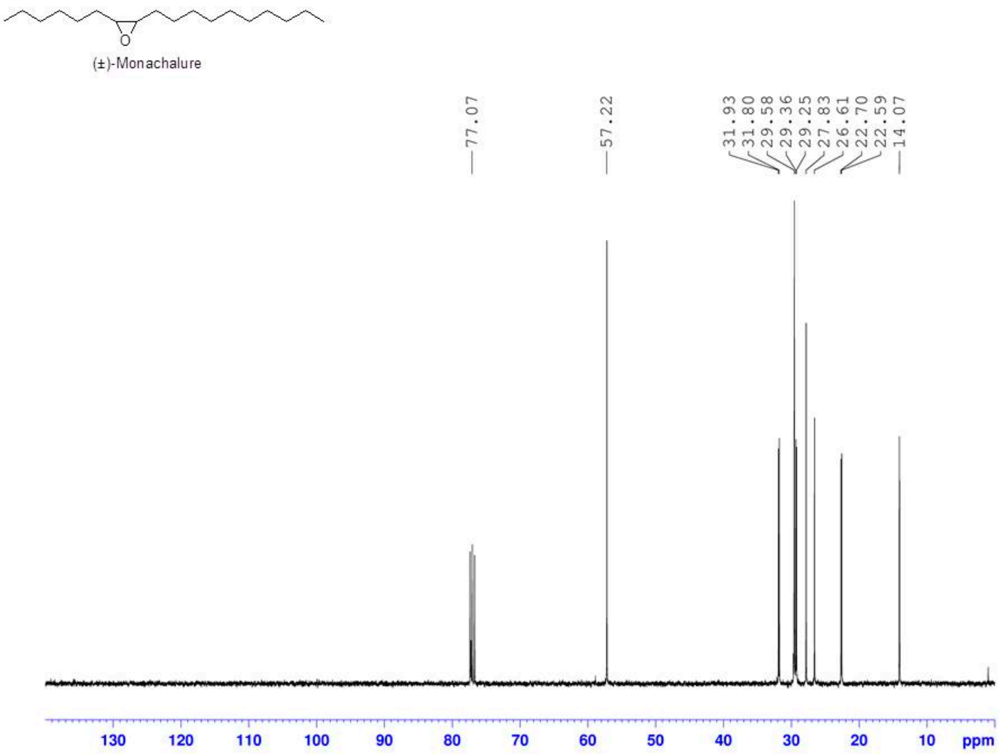

2-Methyl-7-octadecyne (7), S13 ^{1}H-NMR, S14 ^{13}C-NMR * Abbreviated structural formula in ChemDraw.

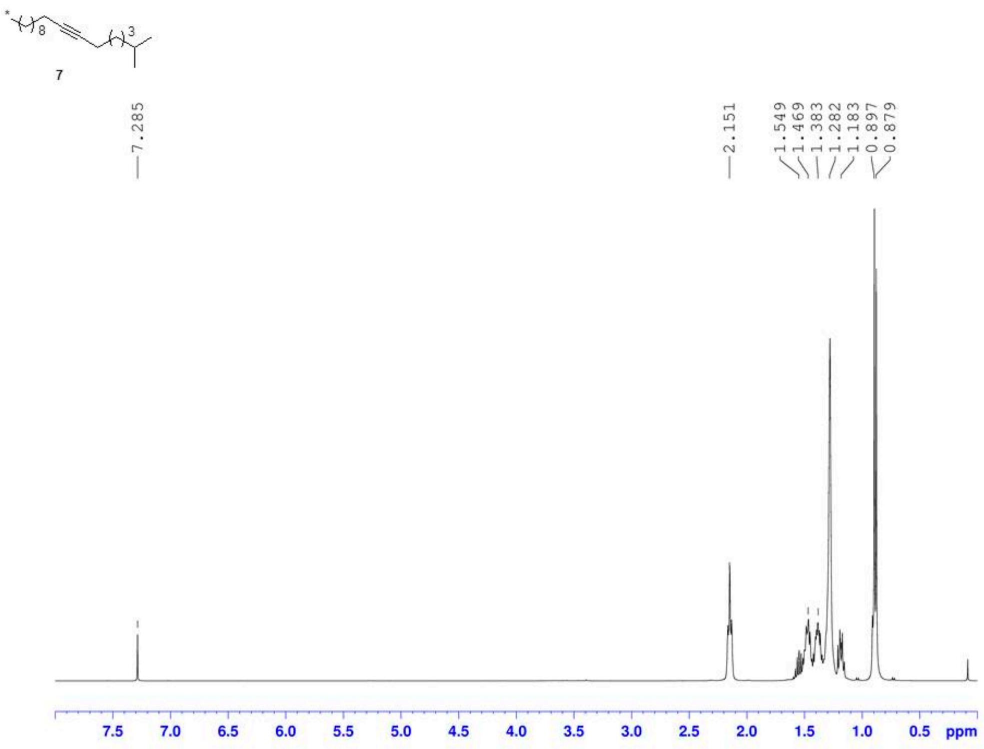

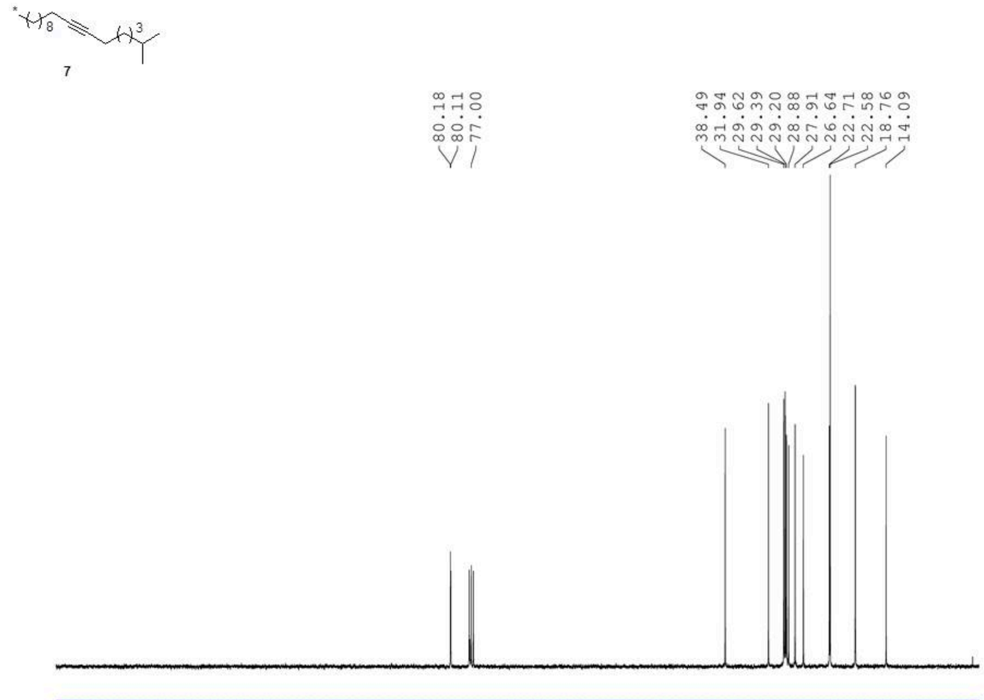

Z-2-Methyl-7-octadecene (9), S15 ¹H-NMR, S16 ¹³C-NMR

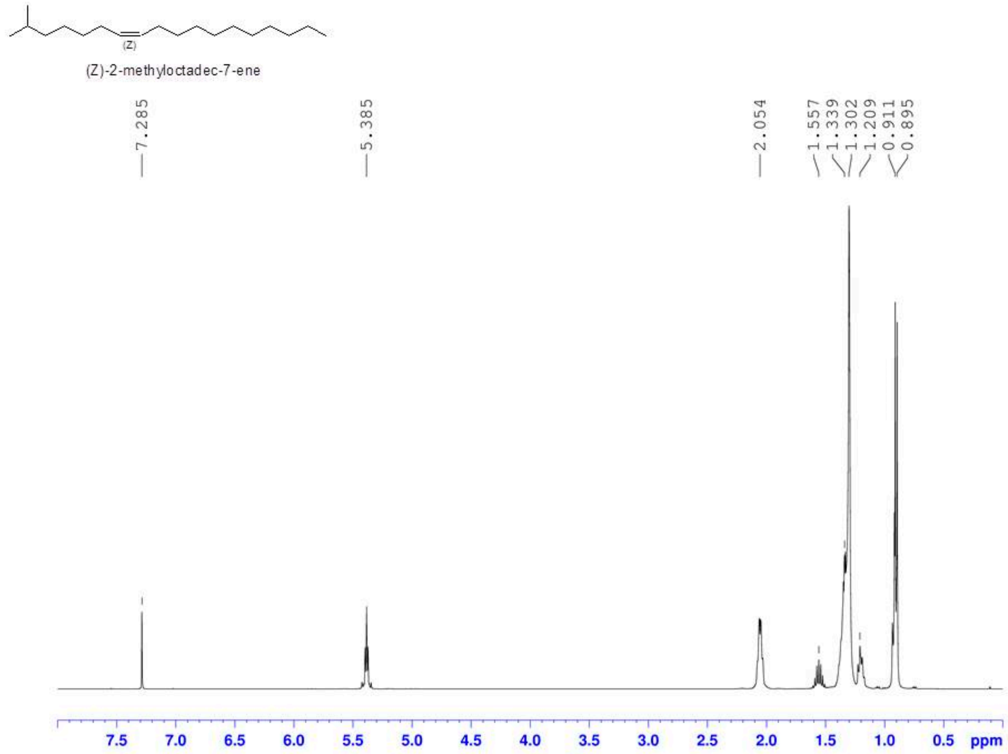

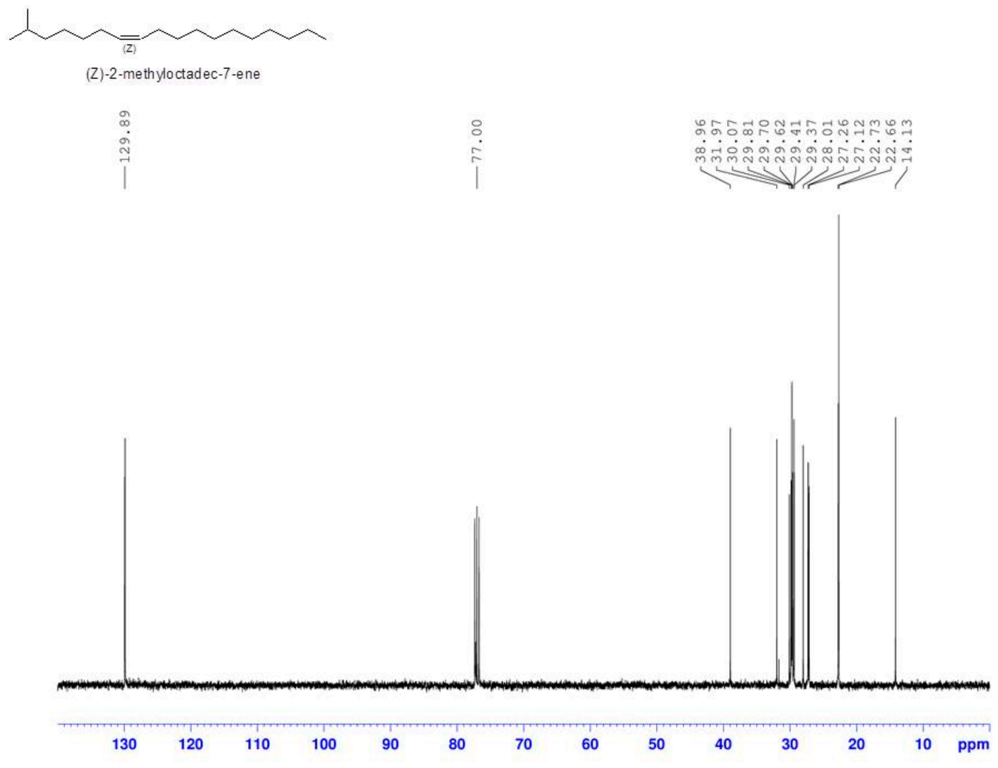

(±)-Disparlure(2), S17 ^{1}H-NMR, S18 ^{13}C-NMR

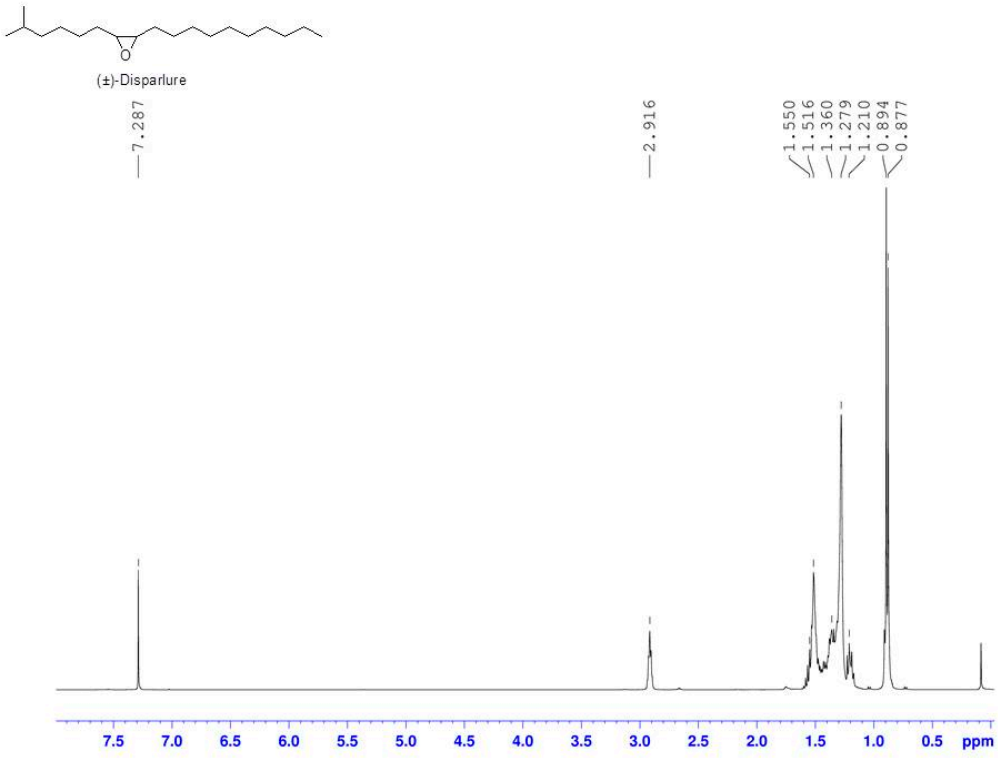
(±)-Disparlure
7.287
2.916
1.550
1.516
1.360
1.279
1.210
0.894
0.877
7.5 7.0 6.5 6.0 5.5 5.0 4.5 4.0 3.5 3.0 2.5 2.0 1.5 1.0 0.5 ppm

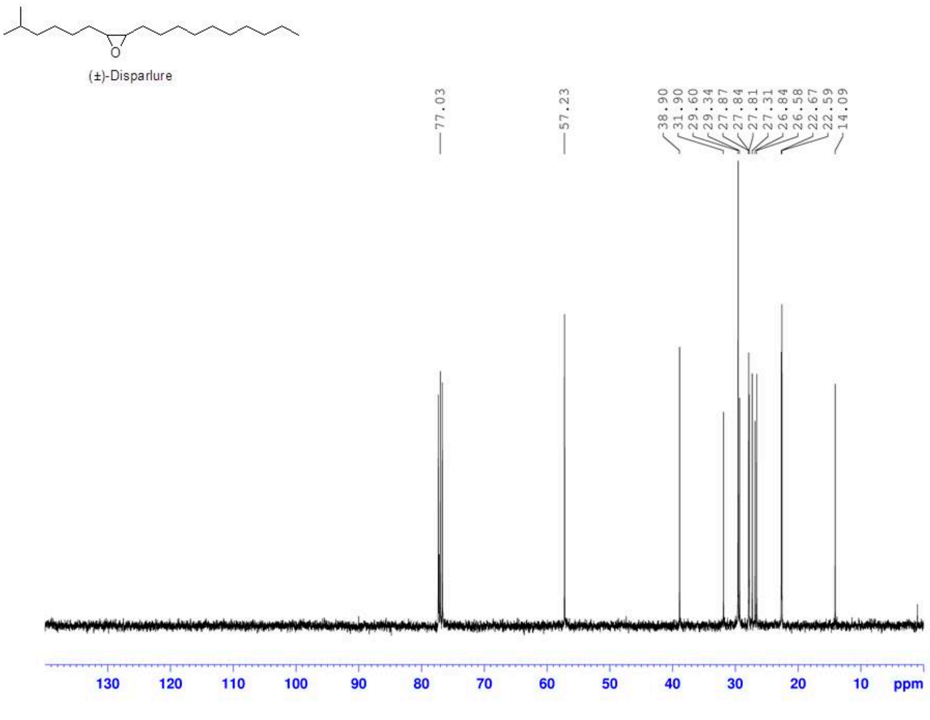
(±)-Disparlure
77.03
57.23
38.90
31.90
29.60
29.34
27.87
27.84
27.81
27.31
26.84
26.58
22.67
22.59
14.09
130 120 110 100 90 80 70 60 50 40 30 20 10 ppm

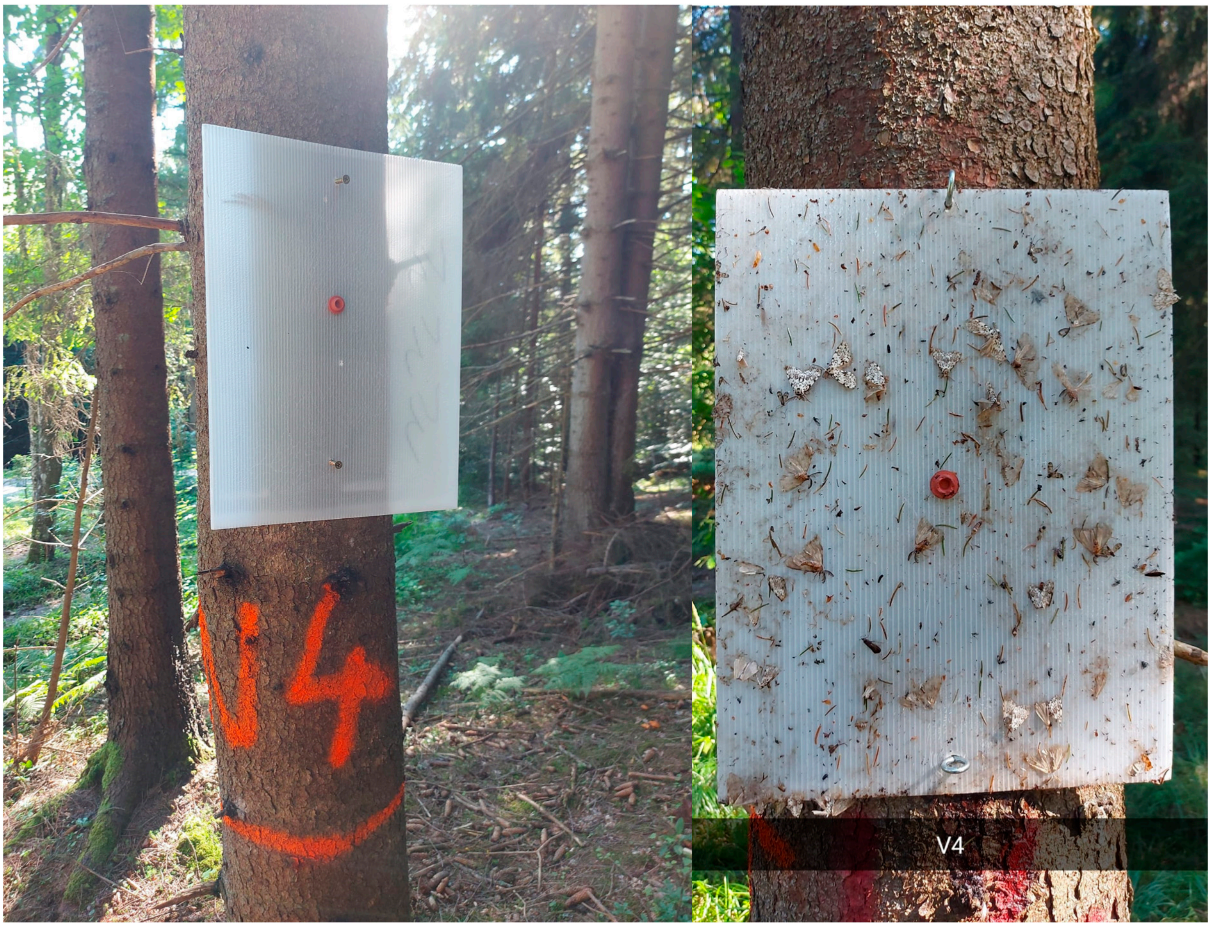

Figure S1. Pheromonal trap atraLYMON, photo Duduman M-L

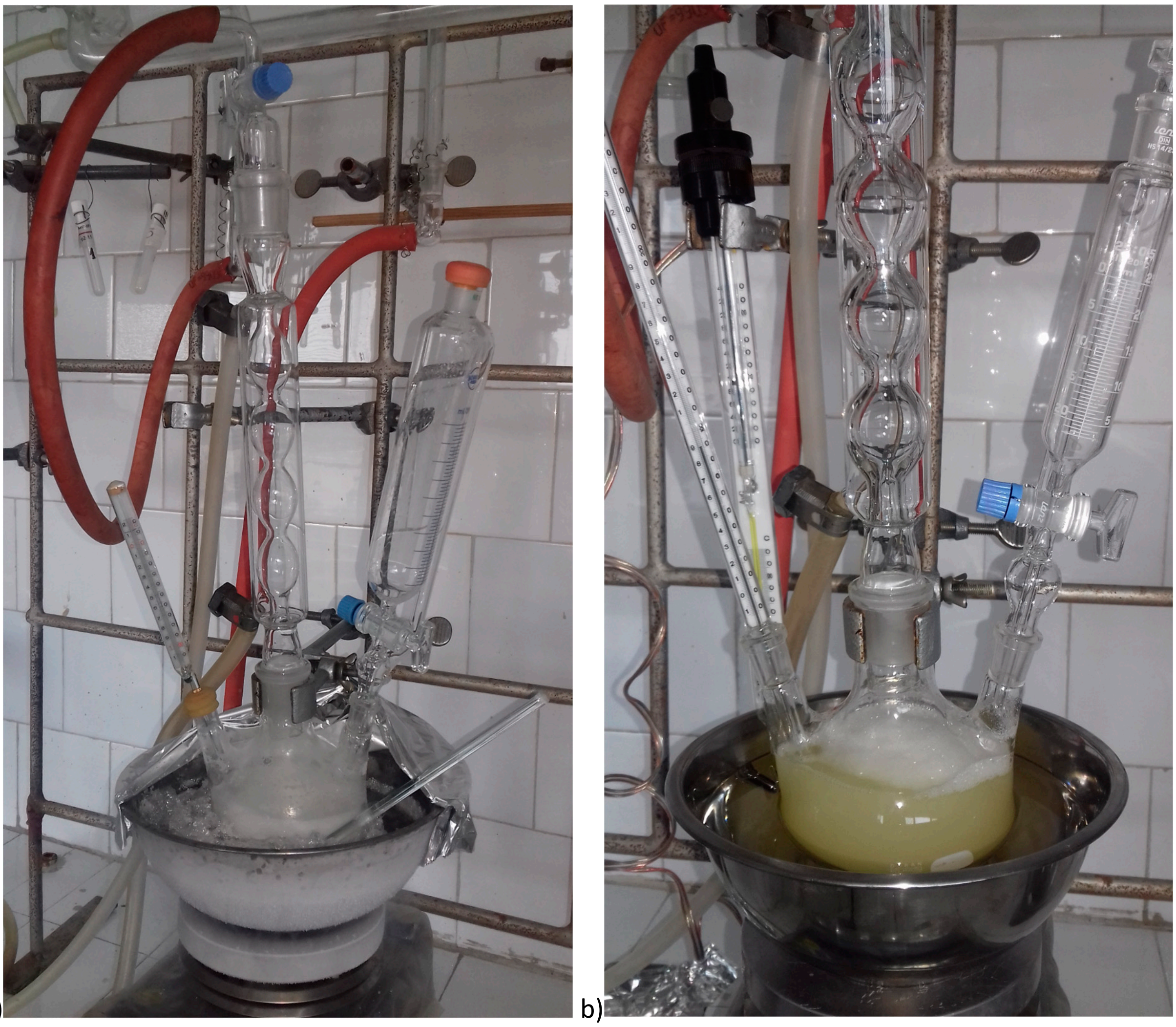

a) b)

Figure S2. Alkylation installation a) Cooling bath, b) heating bath, photo Iuliana Vasian

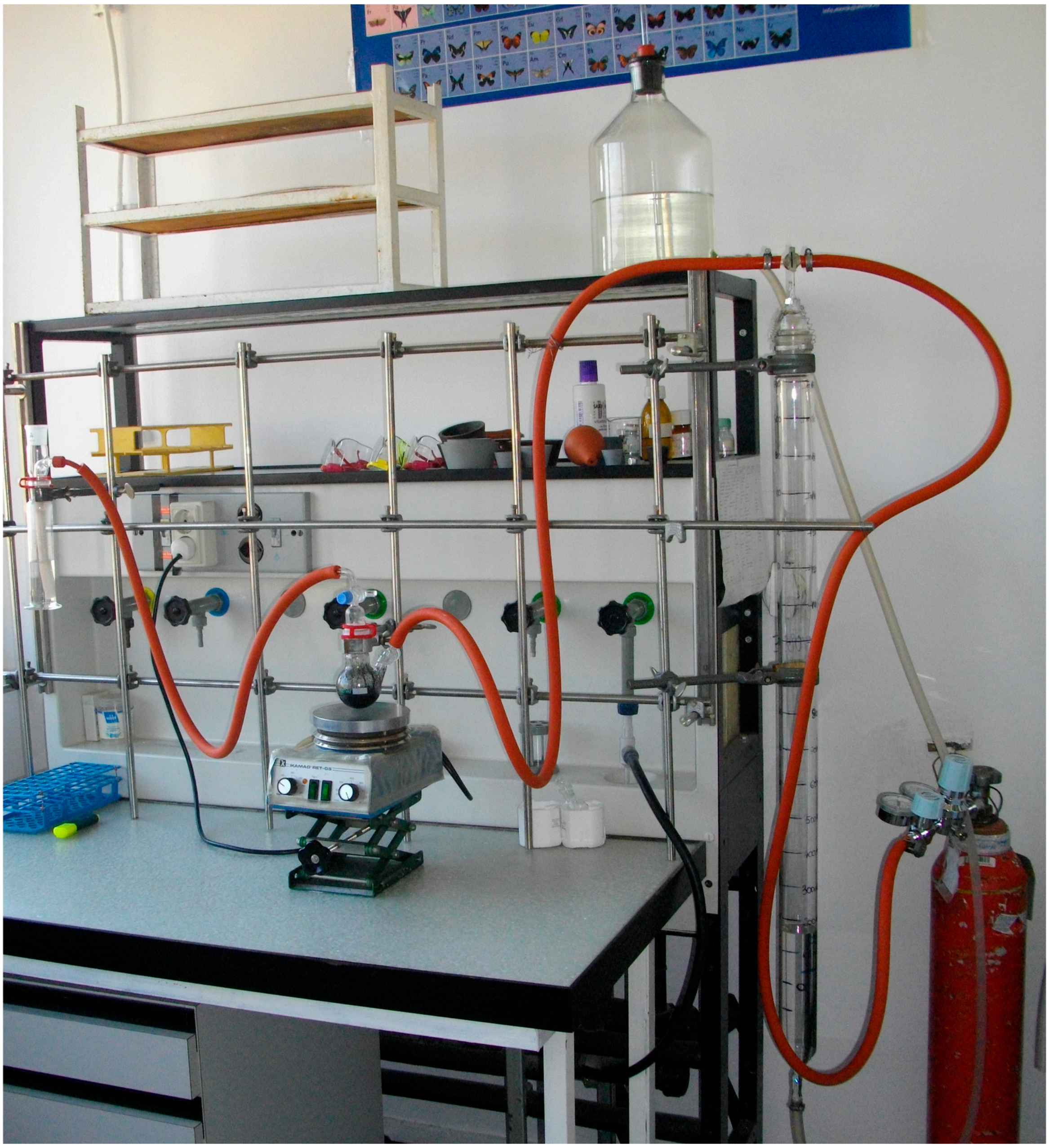

Figure S3. Hydrogenation installation, photo Iuliana Vasian
